# To Correspondents

**Published:** 1841-01-02

**Authors:** 


### TO CORRESPONDENTS.

Our corespondent from Oswego, who asks for the composition of *medicated* wine, refers probably to the *aromatic* wine; an ancient vulnerary remedy, which, although not officinal with us, is largely employed in Europe, and is gradually gaining the reputation it deserves, in this country. The best formula for its preparation is probably the following:

Take, of the dried leaves of the—*Salvia Officinalis*, *Thymus Vulgaris*, *Thymus Serpillum*, *Hyssopus Officinalis*, *Mentha Aquatica*, *Origanum Vulgare*, *Absinthium Officinale*, equal parts, and mix for use.

Take of the mixture four ounces, of port wine and of water each one pint,—macerate for one week, express and filter.

The French Codex directs pure Burgundy wine, and adds two ounces of vulnerary alcohol; the Burgundy is efficiently replaced by diluted port; and the vulnerary alcohol, which adds nothing to the virtues of the preparation, is rejected by most of the French practitioners: it requires for its composition,

The fresh leaves of the *Ocymum Basilicum*, *Melissa Calamintha*, *Hyssopus Officinalis*, *Mentha Piperita*, *Origanum Vulgare*, *Rosmarinus Officin.*, *Satureia Hortensis*, *Salvia Officinalis*, *Thymus Serpillum*, *Thymus Vulgaris*, *Absinthium Officinale*, *Angelica Archangelica*, *Fœniculum Dulce*, *Ruta Graveolens*, the flowers of the *Hypericum Perforata*, *Lavandula Vera*, to be macerated six days in alcohol, and distilled.

The wine itself, without the latter addition, is a most useful and agreeable dressing for indolent and specific ulcers, particularly for chancres.

